# Supplementary material for: Quantitative assessment method for firefighting danger based on numerical simulation of forest fire spread in canyon wind fields
Source: PLoS One. 2025 Jul 24;20(7):e0328733. doi: 10.1371/journal.pone.0328733 (PMC12289084; doi:10.1371/journal.pone.0328733)
Supplement: S1 File — (DOCX) [file pone.0328733.s004.docx]

Supporting Information

The elevation data of the numerical model were obtained from the geospatial data cloud (http://www.gscloud.cn), and the elevation, slope, and aspect data were processed using spatial analysis technology. The land type data were obtained based on the global 30-m fine land cover product (GLC_FCS30-2015) released by Liu’s research team in 2019 . Meteorological data were obtained through online data released by the European Centre for Medium-Range Weather Forecasts.
