# Supplementary material for: Quantitative assessment method for firefighting danger based on numerical simulation of forest fire spread in canyon wind fields
Source: PLoS One. 2025 Jul 24;20(7):e0328733. doi: 10.1371/journal.pone.0328733 (PMC12289084; doi:10.1371/journal.pone.0328733)
Supplement: Attachment — Submitted filename: Response to Reviewers.docx [file pone.0328733.s006.docx]

Dear Editor

In name of all co-authors, it is my pleasure to submit our revised manuscript entitled “Quantitative assessment method for firefighting danger based on numerical simulation of forest fire spread in canyon wind fields” (PONE-D-24-40817).

Dear reviewers, thank you for reviewing our manuscript and providing constructive comments, which greatly assisted us in improving the manuscript. We have substantially revised our experiments. The manuscript has been carefully revised, and a point-by-point response to your comments is listed below. We hope that your concerns have been addressed accurately.

Taken together, we believe that the manuscript has been substantially improved and meets the standards required for publication in PLOS ONE. We look forward to your feedback.

Sincerely,

Dr. Wang

National Institute of Natural Hazards, Ministry of Emergency Management, Beijing, China

**Response to Reviewer #1:
Reviewer’s General comments:**

*In the Methods section, the description of the input data is clearly insufficient. The conceptual risk assessment model does not make sense. Authors should start by conceptually defining the risk model based on the state of the art. This conceptual framework is critical. In order to be able to transform a generic "fire risk" model into a "firefighting risk" model, you must define the indicator classes based on suppression capacity. There are many inconsistencies and even errors in the use of fire science terminology. There are factors that enter the model twice. The way you defined the classes is wrong, it is disconnected from the physical process of fire propagation. It is also unclear how the factor weights were established. You simulated moments of a single fire (why this fire?). You did not describe the fire regime in your study area, nor the fire you used as a reference. You did not include the parameters used in the simulation, nor did you compare the simulated fire with the reference fire. This fire should have only been used to calibrate the parameters required by the simulator. The results should be evaluated using metrics such as overall accuracy, true skill statistics or others (see the work of Pontius on accuracy metrics). Then, these parameters would be used to define simulation scenarios. For each scenario, N fires would be simulated and the AHP would receive fire behavior metrics as input data, as well as the infrastructure frequency reached by the simulated fires.*

*You have a "Results and Discussion" section and a "Discussion" section, but the current discussion has not been done. To what extent does your proposal differ from others? Figures 8 and 9 are not understandable (why was the assessment not carried out for the entire study area?), and once again you are not consistent in terminology (what is "firewire strength"?). In the end, you have little more than a simulation that it is not even possible to know if it was done well.*

*The manuscript is very immature, and is not yet of sufficient quality to be considered for publication.*

**Reply:** We sincerely thank the reviewers for their meticulous evaluation and invaluable feedback, which has guided substantial improvements to our manuscript. In response to your comments, we have implemented comprehensive revisions as follows:

In the Methods section, we provided detailed descriptions of input data to ensure spatial consistency across datasets. The conceptual framework for firefighting risk assessment was restructured with a focus on "firefighter safety risk," incorporating four core indicators—fire behavior, fuel complexity, terrain exposure, and operational exposure—to enhance model design. Terminology inconsistencies were systematically addressed (e.g., replacing "firewire strength" with the more precise term "fireline intensity").

For the fire process analysis, we added an in-depth explanation of the "3.28" Muli fire, clarifying its implications for risk assessment. Figures 8 and 9 now explicitly demonstrate that the actual burned area has undergone a risk assessment, while the unburned area remains untreated.

In the Discussion section, we expanded on the optimization of the wind field model and explored the accuracy of the proposed model. Additionally, we discussed current forest fire suppression methods and their implications for future research.

We deeply appreciate the reviewers' insights, which have significantly enhanced this work and inspired further exploration. We remain open to additional guidance and hope the revised manuscript meets the journal's rigorous standards.

**Detailed comments:**

**Comment 1:** *L108: What do you mean by“climate is complex”?*

**Reply:** Thank you for pointing out this ambiguity. We have revised the sentence to more precisely specify the climatic characteristics of the study area. The original phrase “climate is complex” has been replaced with: “In the research area, the climate is characterized by pronounced seasonal fluctuations and a high frequency of extreme weather phenomena” (Lines 163-164). This revision clarifies the climatic complexity by providing concrete descriptors, such as seasonal variability and the occurrence of extreme weather events, which are directly linked to fire risk.

**Comment 2:** *L109: By "fire protection period" do you mean "fire season"? If so, what period covers the "fire season" in your geographic context?*

**Reply:** We appreciate the suggestion to standardize terminology. The term “fire protection period” has been replaced with the more widely recognized term “fire season,” and the specific months are now explicitly stated: “especially marked by ongoing drought conditions during the fire season, which typically spans from January to May” (Lines 164-165). This adjustment aligns with regional fire management practices in southwestern Sichuan and enhances the clarity and consistency of the manuscript.

**Comment 3:** *L110: Please change“The vegetation types primarily comprise (…)” by “The landscape is mainly covered by (…)”*

**Reply:** Revised as suggested:“The landscape is mainly covered by evergreen coniferous forests...” (Lines 165)

**Comment 4:** *L111: What do you meany by“serious swamping”? Please clarify. Will there be more peat bogs than swamps?*

**Reply:** We apologize for the unclear terminology. The phrase “serious swamping” has been revised to specify the presence of peat bogs:“In addition, interforest meadows featuring remarkable peat bog development are prevalent, increasing the likelihood of underground fires” (Lines 167-168)

**Comment 5:***L112: Please change“underground fire occurrence” by“underground fires”*

**Reply:** Revised as suggested: “increasing the likelihood of underground fires” (Lines 168)

***Comment 6:*** *L115: Change to“Input data and geoprocessing”*

**Reply:** The section title has been updated to: “Input data and geoprocessing” (Lines 172)

**Comment 7:** *L116-120: The description of the input data has to be more detailed. For example, it is important to mention what input data allowed generating the DEM, what methods were used to generate the derivatives that give rise to the slope and aspect (Horn's method?) and what software was used. The designation "land type" is unspecific, apparently it is a land cover map whose metadata must be minimally described (number of classes, method used, input data and auxiliary data that supported the construction of the dataset). Are meteorological data reanalysis? What scale are they on?*

**Reply:** We have expanded the data description to include specific sources，processing methods and rewrote it: “Three fundamental data types were required to implement the forest fire spread and WindNinja models: digital elevation model (DEM) data, land cover data, and meteorological data. The DEM data were sourced from the Geographic Spatial Data Cloud (http://www.gscloud.cn) and then processed using spatial analysis techniques to yield slope and aspect data with a 30-meter spatial resolution. The land cover data were obtained from the global 30-meter spatial resolution fine land cover product (GLC_FCS30-2015) published by Liu Liangyun’s research team in 2019[25]. The meteorological data were collected from the European Centre for Medium-Range Weather Forecasts, using the recorded meteorological data from the nearest weather station to the Muli fire site (station number 56462; 29°N, 101.5°E) to inform the surface average wind field. This study also incorporated satellite imagery from Landsat 8 (http://www.gscloud.cn/) to accurately delineate areas affected by Muli fire. Before integrating this data into the model, it underwent a series of preprocessing steps, including radiometric correction, image registration, image cropping, and image mosaicking, to ensure uniform spatial extent across all datasets.”(Lines 173-185)

**Comment 8:**. *L122: Change the title to “Wildire simulations”*

**Reply:** The revised section title is now: “Wildfire simulations”

**Comment 9:**. *L123: Add a reference of Mark Finney to FARSITE*

**Reply:** A citation to Finney (1998) has been added: “FARSITE(Finney, 1998) is a two-dimensional fire modeling system that serves as a valuable supplementary decision-making instrument for the prevention and management of forest fires.” (Lines 189-190)

**Comment 10:**. *L124: Add "fire" before "spread"*

**Reply:** Revised as suggested: I removed “The Rothermel model and Huygens principle are two crucial theoretical bases of FARSITE that can simulate the spread of various types of fire under complex environmental conditions and obtain the simulation and prediction results of dozens of behavioral characteristic parameters during fire development.” as this was repetitive. This was described in the introduction.

**Comment 11:**. *L124-126: Please rewrite to improve readability*

**Reply:** Revised as suggested: I removed “The Rothermel model and Huygens principle are two crucial theoretical bases of FARSITE that can simulate the spread of various types of fire under complex environmental conditions and obtain the simulation and prediction results of dozens of behavioral characteristic parameters during fire development.” as this was repetitive. This was described in the introduction.

**Comment 12:**. *L123: Add a reference to WindNinja*

**Reply:** A citation to (Forthofer et al., 2014) has been added: “WindNinja (Forthofer et al., 2014) was used to produce high-resolution wind fields by incorporating the DEMs and meteorological data” (Lines 191-192)

**Comment 13:**. *L127-129:"In addition" why? Simulations can be carried out with surface wind simulation or not. This choice is part of the simulation process, not exactly something independent.*

**Reply:** We apologize for the ambiguous phrasing. The revised text clarifies the rationale: “WindNinja (Forthofer et al., 2014) was used to produce high-resolution wind fields by incorporating the DEMs and meteorological data. A significant benefit of WindNinja is its ability to integrate seamlessly with FARSITE, allowing for the direct application of the simulated wind fields.” (Lines 191-193)

**Comment 14:**. *L129-130: Flammap already does this integration without having to import WindNinja outputs*

**Reply:** We selected WindNinja for its superior performance in complex terrain. WindNinja generates spatially varying wind fields compatible with FARSITE.

**Comment 15:**. *L131-138: Move to the previous section and rewrite to improve readability*

**Reply:** The description of landscape file preparation has been moved to the “Comparison and selection of the forest fire behavior index” section and revised for clarity .

**Comment 16:**. *L132: Change“slope”by“slope angle”and“slope direction” by“slope aspect”*

**Reply:** Revised to standard terminology: “elevation, slope angle, and slope aspect”

**Comment 17:**. *L135: What are the "classic fuel model values"? NFFL? Scott & Burgan? The reclassification of land cover to fuel models needs to be better described and should be supported by photographs.*

**Reply:** Fuel models are now explicitly defined using the Scott and Burgan (2005) system: “The assignment of fuel types was conducted based on the 40-fuel model system established by Scott and Burgan (2005), including specific models such as M186 for high-load broadleaf litter.”(Lines 200-201)

**Comment 18:**. *L142: Change“Forest fire behavior encompasses the various changes that occur throughout the fire-spreading process of forest fuel (…)”by“Wildfire behavior encompasses the spatial and temporal dynamics that occur throughout the fire-spreading process across different fuel complexes (…)”*

**Reply:** Revised as suggested: “Wildfire behaviors encompass the spatial and temporal dynamics that occur during the fire-spreading process across different fuel complexes...” (Lines 205-206)

**Comment 19:**. *L143-144: Please be careful in your use of terminology:*

*"spreading speed of the fire head" = "fire rate of spread"*

*"fire intensity" = "fireline intensity"*

*"firefield scope expansion" = ?*

*"other extreme fire behaviors" = ? (probability of crown fire?)*

**Reply:** Revised to standard terminology: “Wildfire behavior is characterized by several factors, including the fire rate of spread, the expansion of the fire perimeter, the escalation of the fireline intensity, and extreme fire behaviors such as spotting, fire whirls, and flare-ups” (Lines 207-209)

**Comment 20:**. *L147: The flame length is directly proportional to the fireline intensity, but not to the fire rate of spread.*

**Reply:** We thank the reviewer for this correction. The sentence has been revised to reflect this relationship:“The spread rate of a wildfire, recognized as a fundamental indicator of fire behaviors, significantly impacts the development and expansion of the fire. Furthermore, it is an essential metric for evaluating the dynamic alterations that occur within the fire environment during firefighting efforts. This spread rate also serves as a primary criterion for fire command personnel when organizing firefighting teams.” (Lines 211-215)

**Comment 21:**. *L154-156: Move to the next section*

**Reply:** The text describing the AHP method has been moved to the “Firefighting risk assessment model” section

**Comment 22:**. *L158-159: You state that“fire behavior, forest fuel factor, topography, and exposure were identified as the four first-level indexes”. How? What other factors did you consider? Topography and fuels are already integrated into fire behavior indicators. You are "doubling" the weight of these factors in the analytical process.*

**Reply:** We sincerely appreciate the reviewer’s insightful critique regarding the selection of first-level indices. We acknowledge that fire behavior inherently incorporates interactions between fuels and topography. However, in designing this firefighting risk assessment model, our goal was to explicitly isolate the unique contributions of each factor to operational safety, rather than relying solely on composite fire behavior metrics. While fuels and topography influence fire behavior, their operational impacts extend beyond combustion physics. For example, a high-slope area with moderate fire intensity may still pose extreme risks due to limited escape routes—a dimension not captured by fire behavior indices alone. By separating these factors, we ensure that suppression capacity and safety are explicitly weighted in the risk calculus.

**Comment 23:**. *L160: You have to maintain consistency in the terminology used*

**Reply:** We apologize for inconsistencies. Terminology has been standardized (e.g., “fuel type” → “fuel model”, “slope” → “slope angle”) throughout the manuscript.

**Comment 24:**. *L161: What do you mean by“secondary evaluation indexes”?*

**Reply:** The term “secondary evaluation indexes” refers to sub-indicators under each first-level index. For clarity, we now use “secondary indicators”.

**Comment 25:**. *L161:“Fuel type”or fuel models?*

**Reply:** Revised to “fuel model” to align with the Scott and Burgan (2005) .

**Comment 26:**. *L161:“Fuel type and intensity”? What’s“fuel intensity”?*

**Reply:** We apologize for the ambiguity. “Fuel intensity” has been replaced with “fuel density”, defined as “Number of plants per unit area”.

**Comment 27:**. *L162: "Slope" and "slope inclination" are basically the same. Maybe you mean "slope angle" and "slope aspect"*

**Reply:** Revised to standard terms: “slope angle and slope aspect”.

**Comment 28:**. *L163-164: The distance to a burnt edge is not exactly a measure of exposure. Infrastructure exposure can be assessed by the frequency of intersections by simulated fires. See the works of Bruno Aparício and Alan Ager.*

**Reply:** We sincerely appreciate the reviewer’s insightful suggestion regarding the assessment of infrastructure exposure through simulated fire intersections, as proposed by Ager et al., and we acknowledge the methodological rigor of this approach. In the context of firefighting risk assessment, our choice to prioritize "distance to burnt edges" as a proxy for exposure was driven by the unique operational challenges and environmental conditions inherent to remote mountainous regions in China, which differ significantly from fire-prone areas in other countries (e.g., North America or Australia). In China’s alpine canyon terrains, firefighting operations often occur in areas with sparse infrastructure (e.g., limited roads, water sources, or settlements). Here, the immediate threat to firefighters stems from rapid fire spread and entrapment risks, where proximity to active fire edges directly impacts escape route viability. Unlike regions with dense infrastructure, the frequency of fire intersections with human assets may be less relevant in such environments, whereas distance to dynamic fire fronts becomes a critical survival metric. We thank the reviewer for this valuable critique, which highlights the importance of context-specific risk modeling. Our approach aligns with the urgent need for adaptive strategies in under-resourced firefighting environments, while laying groundwork for broader applications.

**Comment 29:**. *L171: The scores in Table 1 are anything but comprehensive.*

**Reply:** We sincerely appreciate the reviewer’s feedback on the scoring system in Table 1. As this study is an exploratory effort aimed at developing a preliminary framework for firefighting risk assessment in complex canyon terrains, we intentionally prioritized simplicity and operational relevance over exhaustive comprehensiveness. Our goal was to identify key actionable indicators that could be readily applied in data-scarce environments typical of remote mountainous regions. This exploratory phase serves as a foundational step to validate the integration of wind-fire coupling simulations with risk assessment. Future work will expand the scoring criteria as part of a broader collaboration with local agencies to collect high-resolution data and refine thresholds. We thank the reviewer for underscoring this limitation, which will guide our efforts to balance practicality and comprehensiveness in subsequent studies.

**Comment 30:**. *L171-172: The use of natural breaks is not recommended to define risk classes. Class thresholds must be defined according to the ecological process under evaluation. You have resorted to a purely statistical form of division without any connection to the physical process of fire spread or the fire suppression capacity.*

**Reply:** Thank you for raising this important point concerning the definition of risk classes. We fully concur that ecological and operational thresholds are optimal for risk classification. Nevertheless, in this study, the natural breaks method was employed as an initial step to objectively delineate risk gradients, given the lack of region-specific suppression benchmarks or validated fire behavior thresholds for the Muli area. We sincerely appreciate your critique, which underscores a critical direction for enhancing risk assessment frameworks in data-scarce environments.

**Comment 31:**. *L172:“Fire risk” and“fire danger” are not the same thing. See the seminal works of Hardy, Bachmann or Keane*

**Reply:** We sincerely thank the reviewer for their insightful critique regarding the distinction between "fire risk" and "fire danger" and for directing us to the seminal works of Hardy, Bachmann, and Keane. We fully acknowledge the importance of precise terminology in fire science and have carefully revised the manuscript to align with these foundational definitions. This study explores the dangers of forest fire fighting. Therefore, the term "fire danger" is more appropriate. All instances of "fire risk" have been replaced with "fire danger" where appropriate.

**Comment 32:**. *L173: Change“meteorological”by“fire-weather”*

**Reply:** Revised as suggested: “fire-weather conditions”. (Lines 246)

**Comment 33:**. *L174-175: But you do not present any of the fuel structure indicators that you mention*

**Reply:** Thank you for your attention to the details of our fuel characterization. We recognize the significance of fuel structure indicators (e.g., surface-to-volume ratio, canopy base height) in fire behavior modeling. In this study, however, our main emphasis was on integrating spatially explicit fuel type and density data to achieve a balance between model complexity and computational feasibility. Consequently, fuel structure indicators were not included.

**Comment 34:**. *L176: How have you measured “fuel density”?*

**Reply:** Thank you very much for your careful review and kind comments. We use vegetation cover (FVC) to measure fuel density.

**Comment 35:**. *L176-178: Repeated*

**Reply:** Redundant text has been removed to improve conciseness.

**Comment 36:**. *L184-187: The use of the AHP method is interesting and appropriate, but you do not define how the factor weights were established*

**Reply:** We extend our gratitude to the reviewer for their insightful critique. The AHP weights were derived from expert surveys: “The AHP weights were obtained through expert surveys that assessed pairwise comparisons of the indices. The consistency ratios, verified by Saaty’s methodology, were assessed as less than 0.1”.

**Response to Reviewer #2:
Reviewer’s General comments:** This manuscript constructs a forest firefighting risk assessment model to quantify comprehensive risk based on forest fire behavior, forest fuel, topography, and exposure indexes. The authors also demonstrate the potential of using FARSITE and WindNinja to provide fire behavior information for their risk assessment model.

**Reply:** We sincerely appreciate your significant comments on our manuscript, which have been highly instrumental in helping us enhance the quality of our work. We have made every effort to revise the manuscript in accordance with your valuable suggestions.

**Detailed comments:**

**Comment 1:** *L52-53:* T*he authors adopted FARSITE as the fire spread model in this study, yet it was not mentioned in the literature review section. I suggest enhancing the literature review to further discuss various fire spread models.*

**Reply:** Thank you for this critical observation. We have expanded the literature review to incorporate a detailed discussion of fire spread models, with particular emphasis on FARSITE. The revised text now reads as follows:

“FARSITE integrates the Rothermel surface fire spread model using fundamental physical principles and empirical data. FARSITE uses this principle to break down a fire perimeter into discrete segments ("wavelets") that propagate independently based on local environmental conditions. It computes fire spread rates by considering various factors such as fuel type, meteorological conditions, and terrain slope, achieving a high degree of accuracy through the application of an energy balance equation[10]. In contrast to more intricate models that rely specifically on physical processes, such as FIRETEC and WFDS, FARSITE is more adept at rapidly simulating large-scale fires while maintaining manageable scientific and computational costs. Sibanda et al. used the FARSITE and WindNinja models to simulate forest fire behaviors in the challenging topography of Nepal, aiming to estimate carbon emissions[11]. Owing to the prolonged simulation times associated with extensive fire areas, Sanjuan et al. proposed a zoning strategy for local wind field calculations, thereby optimizing wind field simulations within time constraints[12]. Seungmin et al. improved the accuracy of FARSITE’s fire spread predictions by integrating the directional spread rate adjustment factors and genetic algorithms. These studies have contributed to the development of more effective fire response strategies, which can enhance resource allocation and mitigate the overall impact of fires.”(Lines 69-83)

**Comment 2:** *L91-97: The research question and unique contribution are not explicitly stated. I recommend revising this paragraph to clearly articulate the research question and contributions.*

**Reply:** We sincerely appreciate the suggestion to further clarify our research focus. The introduction now explicitly states:

“Considering the unique geographical characteristics and prevalent fire incidents in southwest Sichuan, the aim of this study is to investigate the following research question: In what ways can a coupled WindNinja–FARSITE model, along with a comprehensive danger assessment model, enhance the quantification of firefighting dangers in canyon terrains? To achieve this objective, we propose the following substantial contributions:

(1)Developing a high-resolution wind–fire coupling simulation framework tailored to canyon topography.

(2)Introducing an innovative firefighting danger assessment model that integrates fire behaviors, fuel characteristics, terrain features, and exposure indices.

(3)Quantitatively identifying high-danger zones and vegetation types to inform and optimize firefighting strategies.”(Lines 127-137)

**Comment 3:** *L116-120: The topographic and fuel data were extracted from different sources. It would be helpful to mention if the resolution is consistent or if re-sampling was used. What is the resolution used in this study?*

**Reply:** We sincerely thank the reviewer for drawing attention to this gap. The revised methods section now clarifies:

“Three fundamental data types were required to implement the forest fire spread and WindNinja models: digital elevation model (DEM) data, land cover data, and meteorological data. The DEM data were sourced from the Geographic Spatial Data Cloud (http://www.gscloud.cn) and then processed using spatial analysis techniques to yield slope and aspect data with a 30-meter spatial resolution. The land cover data were obtained from the global 30-meter spatial resolution fine land cover product (GLC_FCS30-2015) published by Liu Liangyun’s research team in 2019[25]. The meteorological data were collected from the European Centre for Medium-Range Weather Forecasts, using the recorded meteorological data from the nearest weather station to the Muli fire site (station number 56462; 29°N, 101.5°E) to inform the surface average wind field. This study also incorporated satellite imagery from Landsat 8 (http://www.gscloud.cn/) to accurately delineate areas affected by Muli fire. Before integrating this data into the model, it underwent a series of preprocessing steps, including radiometric correction, image registration, image cropping, and image mosaicking, to ensure uniform spatial extent across all datasets.” .(Lines 173-185)

**Comment 4:**. *L131: More information could be provided for the simulated March 28 Muli Forest fire, such as crown fire activity, burning periods, simulation duration, and fuel moisture.*

**Reply:** We sincerely apologize for the oversight. Additional details have now been included:

“On March 28, 2020, at 19:30, a remarkable forest fire was ignited at the border of Chutouwan Village in Qiaowa Town and Xiangjiao Village in Xiangjiao Township within Muli Tibetan Autonomous County. The area affected by the fire was approximately 230 hectares. On the afternoon of March 29, 2020, a sudden increase in wind speed caused the surface fire to escalate into a crown fire, thereby complicating firefighting efforts. On March 30 and 31, strong winds impeded the effectiveness of the firefighting operations. In the early hours of April 1, firefighting teams capitalized on favorable conditions characterized by low temperatures, high humidity, and minimal wind to combat the blaze. However, in the afternoon of April 1, the northern perimeter of the fire reignited, and under the influence of extreme wind variability, the fire rapidly spread to the northeast and northwest. From April 2 to 6, firefighting teams undertook a comprehensive operation in the affected area, successfully extinguishing the forest fire by April 7” (Lines 150-160).

**Comment 5:**. *L135: Assuming the authors classified fuels based on the 40 Scott and Burgan model, it would be helpful to mention this and cite accordingly.*

**Reply:** We appreciate the opportunity to clarify this. The revised text states: “The assignment of fuel types was conducted based on the 40-fuel model system established by Scott and Burgan (2005), including specific models such as M186 for high-load broadleaf litter.”(Lines 200-201)

**Comment 6:**. *L171-173: Further elaboration of the score assignment is needed. How were the breakpoints decided, and how were those characteristics transferred to scores?*

**Reply:** We sincerely appreciate this important critique. We have enhanced methodological transparency as follows:
“Scores were allocated based on the thresholds derived from historical firefighting incident data and expert assessments, with reference to "Forest and Grassland Firefighting Safety Studies" by Shu et al. For instance, flame lengths exceeding 3 meters (indicative of extreme danger) were assigned the highest score owing to their association with firefighter fatalities.” (Lines 242-246)

Lastly but importantly, we sincerely acknowledge the valuable comments and suggestions provided by the Editor and Reviewers, which have significantly contributed to enhancing the quality of our manuscript. Following the revision and supplementation based on your insightful feedback, we believe that our revised paper now aligns with your expectations. Therefore, we hope that our updated work will receive your support and recognition during the subsequent refereeing process. We look forward to your favorable response. Thank you very much for your attention to our paper.

With best wishes,

Sincerely yours,

Ao Wang , Chenghu Wang , Guiyun Gao , Ningyu Wu , Haiyan Su
