# Supplementary material for: Quantitative assessment method for firefighting danger based on numerical simulation of forest fire spread in canyon wind fields
Source: PLoS One. 2025 Jul 24;20(7):e0328733. doi: 10.1371/journal.pone.0328733 (PMC12289084; doi:10.1371/journal.pone.0328733)
Supplement: Attachment — Submitted filename: Response to Reviewers0703.docx [file pone.0328733.s007.docx]

Dear Editor

In name of all co-authors, it is my pleasure to submit our revised manuscript entitled “Quantitative assessment method for firefighting danger based on numerical simulation of forest fire spread in canyon wind fields” (PONE-D-24-40817R1).

Dear reviewers, thank you for reviewing our manuscript and providing constructive comments, which greatly assisted us in improving the manuscript. We have substantially revised our experiments. The manuscript has been carefully revised, and a point-by-point response to your comments is listed below. We hope that your concerns have been addressed accurately.

Taken together, we believe that the manuscript has been substantially improved and meets the standards required for publication in PLOS ONE. We look forward to your feedback.

Sincerely,

Dr. Wang

National Institute of Natural Hazards, Ministry of Emergency Management, Beijing, China

**Response to Reviewer #3:**

**Detailed comments:**

**Comment 1:** *Only small comment, Keywords: It is crucial to revise the keywords, ensuring they are spelled correctly and avoid general, abbreviations, and plural terms and multiple concepts (avoid, for example, 'and', 'of'). This will help to maintain the precision and clarity of the manuscript.*

**Reply:** *Thank you for this valuable suggestion to enhance keyword precision. We have carefully refined the keywords as follows:*

*Forest firefighting danger; Canyon wind field; Fire spread simulation; FARSITE; WindNinja; Risk assessment*

**Comment 2:** *In the main text, many numeric data are given too many significant figures; two significant figures suffice, and three suffice if the first significant figure is "1."*

**Reply:** *We sincerely appreciate this meticulous observation. We have now comprehensively adjusted numerical values throughout the manuscript:*

**Table 2. Weights of forest firefighting danger assessment index.**

| **Primary index** | **Primary index weight** |
| --- | --- |
| Forest fire behavior | 0.28 |
|  |  |
|  |  |
| Fuel factor | 0.48 |
|  |  |
| Terrain factor | 0.17 |
|  |  |
| Exposure | 0.06 |
| **Secondary index** | **Secondary index weight** |
| Spread rate | 0.61 |
| Fireline intensity | 0.30 |
| Flame length | 0.09 |
| Fuel type | 0.83 |
| Fuel density | 0.17 |
| Slope angle | 0.83 |
| Slope aspect | 0.17 |
| Distance to town | 0.25 |
| Distance to the water source | 0.75 |

**Table 3. Comparison of simulation accuracy of different wind fields.**

| **Wind field** | **Time** | **Area of coincidence area /km^2^** | **Over-simulated area /km^2^** | **Unsimulated area /km^2^** | **SC coefficient** |
| --- | --- | --- | --- | --- | --- |
| Average surface wind field | March 29, 19:00 | 60 | 19 | 6.1 | 0.82 |
|  | March 30, 19:00 | 86 | 59 | 10.8 | 0.71 |
|  | April 1, 19:00 | 78 | 204 | 22 | 0.40 |
| WindNinja wind field | March 29, 19:00 | 62 | 4.4 | 3.9 | 0.94 |
|  | March 30, 19:00 | 86 | 36 | 12 | 0.78 |
|  | April 1, 19:00 | 80 | 178 | 21 | 0.44 |

**Comment 3:** *You must provide all the figures in high resolution and make the labels and legends more legible.*

**Reply:** *We gratefully acknowledge this important recommendation. To improve visual clarity:*

*All figures regenerated at 600 dpi resolution.*

*Enhanced color contrast in categorical displays.*

*Supplementary png files provided.*

*Specific improvements:*

**Fig 1. Assessment model of forest firefighting danger level.**

**Fig 2. Wind field data from March 25 to April 3, 2020 in the research area.**

**Fig 3. Comparison between simulated wind field and actual wind field.**

**Comment 4:** *Conclusion: The findings could be further developed; the article contains a lot of interesting data.*

**Reply:** *We deeply appreciate your encouraging feedback on our data. The conclusion has been substantially enriched:*

*To address the limitations of conventional forest fire spread models for predicting fire behavior in complex terrains such as mountainous and valley regions, this study introduces a coupled simulation approach that integrates the FARSITE forest fire spread simulation system with the high-resolution wind field model WindNinja. Furthermore, a forest firefighting danger assessment model was developed, incorporating factors such as forest fire behavior, fuel characteristics, topographical features, and exposure indices. This model was used to quantify the firefighting danger associated with the Muli forest fire that occurred on March 28 in Liangshan, Sichuan Province. The key findings, significantly enriched by the detailed simulation data, offer a robust scientific foundation for implementing targeted forest firefighting strategies and address the core research question on enhancing danger quantification in canyon terrains:*

*(1)The wind field within the canyon displays a significant diurnal variation, with maximum wind speeds generally recorded between 2 PM and 4 PM. A comparative assessment of the simulated wind speeds against actual local meteorological conditions revealed inconsistencies, with an average deviation of approximately 2 m/s in the simulated data. However, the wind direction exhibits a degree of stability, predominantly blowing from the southwest (SW) and west-southwest (WSW). The high-resolution wind field data produced by the WindNinja model demonstrates its efficacy in accurately simulating the dynamics of forest fire propagation in relation to the canyon's topographical characteristics.*

*(2) The simulation indicated concerning levels of fire behavior risk, with regions categorized as having high and extremely dangerous fire spread rates accounting for 16% and 54% of the study area, respectively, primarily located in the central and southern regions. Although areas exhibiting high-danger fire line intensity were notable at 5.9%, extreme-danger intensity zones, while less prevalent at 0.14%, pose significant flashpoint hazards. Additionally, high and extreme-danger flame lengths encompassed 21% and 8.1% of the area, respectively, demonstrating a strong spatial correlation with high spread rates. This quantitative spatial mapping of zones exhibiting extreme fire behavior represents a significant finding of the integrated model.*

*(3) The analysis of fuel types has identified grasslands, deciduous broadleaf forests, and evergreen coniferous forests as the primary combustible materials. Notably, grasslands demonstrated the highest average fire behavior index (11) and an overall firefighting danger index (10.1), followed by deciduous broadleaf forests with indices of 10.3 and 8.4, respectively. These values significantly surpass the danger levels associated with evergreen coniferous forests, which recorded indices of 7.4 and 6.0. This finding highlights the considerable risk posed by grassland fuels in the region. Additionally, exposure analysis revealed that the threat of firefighting danger escalates with increasing distance from urban areas or water sources.*

*(4) Upon synthesizing all relevant factors, the thorough danger assessment indicated that a significant 37% of the Muli fire site area is subjected to high or extremely high levels of firefighting danger, with concentrations predominantly located in the central and western regions. An analysis based on forest fuel types clearly identified grassland ecosystems as the vegetation type with the highest associated risk.*

*At the Muli fire site, 38% of the area is classified as having a high or extremely high danger of fire, with this danger predominantly located in the central and western regions of the site. An examination of forest fuel classifications reveals that grassland ecosystems represent the highest-risk vegetation type, exhibiting a comprehensive danger index of 10.1. Areas classified as extreme danger are predominantly located in central and western canyons, where southwesterly winds exceeding 18 m/s have been shown to increase fire spread rates by 54%. To reduce the risk of firefighter casualties, we propose the following recommendations: (1) The real-time integration of WindNinja-FARSITE forecasts into firefighting operations. (2) The implementation of preemptive fuel reduction strategies in grasslands situated within a 5 km radius of urban areas. (3) The tactical avoidance of leeward slopes during peak diurnal wind periods, specifically between 14:00 and 16:00.*

**Response to Editor:**

*Thank you for raising this important consideration regarding image copyright. We would like to clarify that all cartographic representations in our study were generated through original processing of open-access Landsat base data (Landsat: http://landsat visibleearth.nasa.gov/). At the same time, we have made the following adjustments to the picture：*

*Fig 4. WindNinja wind field and fire line intensity coupling simulation.*

*Fig 5. Simulation results of fire behavior and exposure in the research area.*

*Fig 6. Forest fire comprehensive danger level map.*

We are deeply grateful for your insightful suggestions, which have significantly strengthened our work. All modifications have been meticulously implemented, with tracked changes provided for your convenience. Should any further clarifications be needed, we remain available to address them promptly.

With best wishes,

Sincerely yours,

Ao Wang , Chenghu Wang , Guiyun Gao , Ningyu Wu , Haiyan Su
